# The 5′ Flanking Region and Intron1 of the Bovine Prion Protein Gene (*PRNP*) Are Responsible for Negative Feedback Regulation of the Prion Protein

**DOI:** 10.1371/journal.pone.0032870

**Published:** 2012-03-07

**Authors:** Guangai Xue, Yoko Aida, Takashi Onodera, Akikazu Sakudo

**Affiliations:** 1 Department of Molecular Immunology, School of Agricultural and Life Sciences, University of Tokyo, Bunkyo-ku, Tokyo, Japan; 2 Viral Infectious Diseases Research Unit, RIKEN, 2-1 Hirosawa, Wako, Saitama, Japan; 3 Laboratory of Biometabolic Chemistry, School of Health Sciences, Faculty of Medicine, University of the Ryukyus, Nishihara, Okinawa, Japan; University of Massachusetts Medical, United States of America

## Abstract

Transcription factors regulate gene expression by controlling the transcription rate. Some genes can repress their own expression to prevent over production of the corresponding protein, although the mechanism and significance of this negative feedback regulation remains unclear. In the present study, we describe negative feedback regulation of the bovine prion protein (PrP) gene *PRNP* in Japanese Black cattle. The PrP-expressing plasmid pEF-boPrP and luciferase-expressing plasmids containing the partial promoter fragment of *PRNP* incorporating naturally occurring single-nucleotide or insertion/deletion polymorphisms were transfected into N2a cells. Transfection of pEF-boPrP induced PrP overexpression and decreased the promoter activity of *PRNP* in the wild-type haplotype (23-bp Del, 12-bp Del, and −47C). Reporter gene assays further demonstrated that the 12- and 23-bp Ins/Del polymorphisms, which are thought to be associated with Sp1 (Specific protein 1) and RP58 (Repressor Protein with a predicted molecular mass of 58 kDa), in intron1 and the upstream region, respectively, and an additional polymorphism (−47C→A) in the Sp1-binding site responded differently to PrP overexpression. With the −47C SNP, the presence of the Del in either the 23-bp Ins/Del or the 12-bp Ins/Del allele was essential for the negative feedback caused by PrP overexpression. Furthermore, deletion mutants derived from the wild-type haplotype showed that nucleotides −315 to +2526, which include the 5′-flanking region and exon1, were essential for the response. These results indicate that certain negative feedback response elements are located in these sequences, suggesting that regulation by transcription factors such as Sp1 and RP58 may contribute to the negative feedback mechanism of *PRNP*.

## Introduction

Prion diseases are neurodegenerative disorder transmitted by prion infection, which is attributed to ingestion of proteinaceous particles into normal brain, leading to accumulation of an abnormally folded form of the prion protein (PrP^Sc^) as a consequence of the conformational conversion of endogenous cellular prion protein (PrP^C^) [1], [2]. Expression of prion protein (PrP) is necessary for transmission, because PrP gene-knockout mice are resistant to infection [3] and conditional knockout during disease progress results in the cessation of observed behavioural changes and neuronal loss [4]. In contrast, increased expression in animal models is associated with increased susceptibility and shortened incubation time [5]. Therefore, the presence/absence and/or the level of PrP^C^ expression seem to be critical for pathogenesis of prion diseases [6], [7].

Some genes have regulatory systems for maintaining the corresponding intracellular protein concentration. These systems prevent overexpression of the gene by repressing its own expression in a dose-dependent manner [8]. In this case, excess protein downregulates activity of the promoter, thereby decreasing protein production and controlling expression at a constant level. This type of control mechanism is known as “negative feedback regulation”.

Here, we examined whether there is a negative feedback regulatory system to control the expression of bovine PrP gene (*PRNP*) by analyzing the promoter activity of *PRNP* under the exogenous overexpression of PrP. To investigate the influence of PrP overexpression on the *PRNP* promoter, a bovine *PRNP* promoter luciferase vector and a bovine PrP expression vector were co-transfected into N2a cells. The mechanism by which PrP overexpression influences the *PRNP* promoter were further examined by using six segregated haplotypes containing single nucleotide polymorphisms (SNPs) in exon1, intron1 and the 5′ flanking region of *PRNP*, as well as the corresponding deletion mutants.

## Results

To examine the absolute transfection efficiency, N2a cells were transfected with a green fluorescent protein (GFP) expression vector (Fig. S1). Forty-eight hours after transfection, the cells were analyzed by fluorescence microscopy. A transfection efficiency of approximately 80% was observed. After determining the absolute transfection efficiency, 2.5 µg, 3.75 µg, 5 µg or 10 µg of pEF-boPrP or the empty vector pEF-BOS was transfected into N2a cells. Forty-eight hours after transfection, the level of PrP expression was measured by sandwich ELISA using a combination of T2 as a capture mAb and 1D12-HRP as a detection mAb. The expression level of PrP in N2a cells after transfection with 5 µg or 10 µg of pEF-boPrP was higher than that in untransfected cells or cells transfected with empty vector cells (Fig. S2).

**Figure 1:**
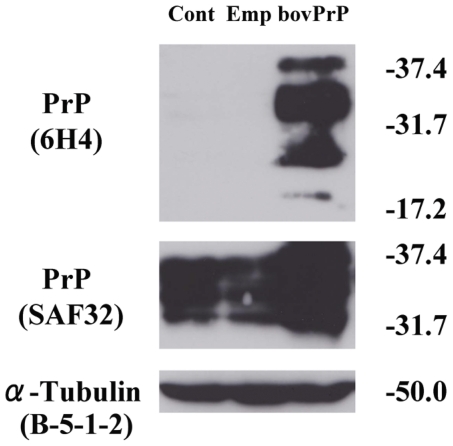
Expression of PrP in pEF-boPrP-transfected N2a cells. The bovine PrP expression vector pEF-boPrP was transfected into N2a cells. Forty eight hours after transfection, cells were lysed, and then the proteins were separated by 12% SDS-PAGE and blotted. The resulting blots were analyzed using anti-PrP mAb 6H4 as a specific antibody for bovine PrP protein, SAF 32 for PrP of all species, or B-5-1-2 for α-tubulin as an internal control.

Subsequently, to further confirm the expression of PrP, cell lysates were prepared 48 h after transfection with 5 µg of pEF-boPrP or pEF-BOS. As shown in Fig. 1, three strong bands with a molecular mass between 17.2 kD and 37.4 kD were observed in the pEF-boPrP-transfected lysate, but not in lysates from cells transfected with the empty vector (pEF-BOS) or untransfected cell lysates (upper panel) by Western blotting using 6H4, an anti-PrP antibody that recognizes bovine PrP but has a weak reaction to mouse PrP. Similarly, by using SAF32, an anti-PrP antibody that strongly recognizes both bovine and mouse PrP, overexpression of bovine PrP in pEF-boPrP-transfected cells was confirmed. Relatively similar expression levels of α-tubulin (internal control) were detected, indicating that protein concentrations were similar in each group. Taken together, these results suggest that N2a cells transfected with pEF-boPrP (5 µg) could overexpress bovine PrP.

**Figure 2:**
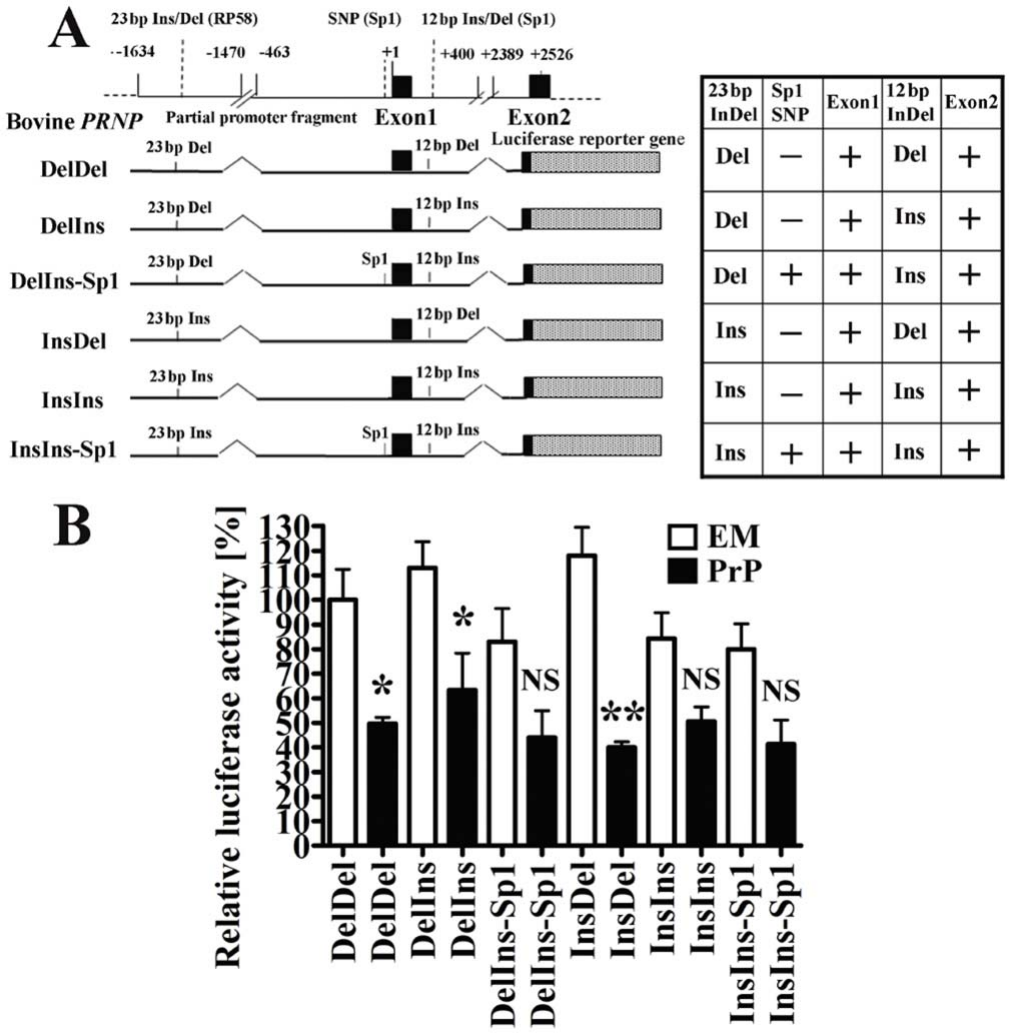
SNP constructs show that the 23-bp deletion in the upstream region of *PRNP* and/or a 12-bp deletion in intron1, coupled with the absence of the Sp1 SNP and the presence of exon1, are required for the negative feedback response to PrP overexpression. (A) Map of the portion of bovine *PRNP* containing the 5′-flanking region and exons 1 and 2 is shown on the top line. Dotted box = Luciferase gene; black boxes = exon 1 and exon 2, which include numbers denoting the position of the reported transcription start site (+1) of the *PRNP* promoter region. The 23-bp indel, 12-bp indel, and SNP regions are also indicated above the reporter gene constructs. The absence (−) and presence (+) of each region in the reporter gene constructs are shown in the Table on the right. (B) Graph representing the relative luciferase activities obtained with the above reporter plasmids in the presence of either an empty vector, pEF-BOS (EM, open bars), or pEF-boPrP (PrP, solid bars). The pGL3-Control vector (with the standard SV40 promoter) was used for normalization between different experiments (relative light units (*RLU*) = (firefly luciferase_construct_/*total protein*_construct_)/(firefly luciferase_control_/*total protein*_control_)). Relative luciferase activities (Mean ± S.D.) for 3 replicate experiments were compared with that of the pGL3-control plasmid (1%). A significant difference of luciferase activity in pEF-boPrP-transfected cells as compared with corresponding empty vector-transfected cells is shown by one asterisk (*, <0.05) or two asterisks (**, <0.01). NS indicates no significant difference.

To investigate the effect of PrP overexpression on the bovine *PRNP* promoter region, the bovine *PRNP* promoter luciferase vector and pRL-SV internal control plasmid, as well as the bovine PrP expression vector pEF-boPrP or empty vector pEF-BOS, were co-transfected into N2a cells (Fig. 2). All constructs tested in the forward orientation exhibited promoter activity, as indicated by the induction of luciferase reporter gene expression in N2a cells (Fig. 2A). The activity of cells transfected with pGL3-control plasmid was taken as 1%. Under basal conditions (i.e., cells transfected with empty vector) DelDel, DelIns, InsDel had higher luciferase activity compared to DelIns-Sp1, InsIns, and InsIns-Sp1 (Fig. 2B, white bars). Overexpression of PrP significantly inhibited DelDel and DelIns promoter activities in the absence of Sp1-SNP, whereas InsDel promoter activity was strongly inhibited by PrP overexpression. However, InsIns in the absence and presence of Sp1-SNP, and DelIns and InsIns in the presence of Sp1-SNP (DelIns-Sp1 and InsIns-Sp1) showed no significant changes on overexpression of PrP (Fig. 2B).

**Figure 3:**
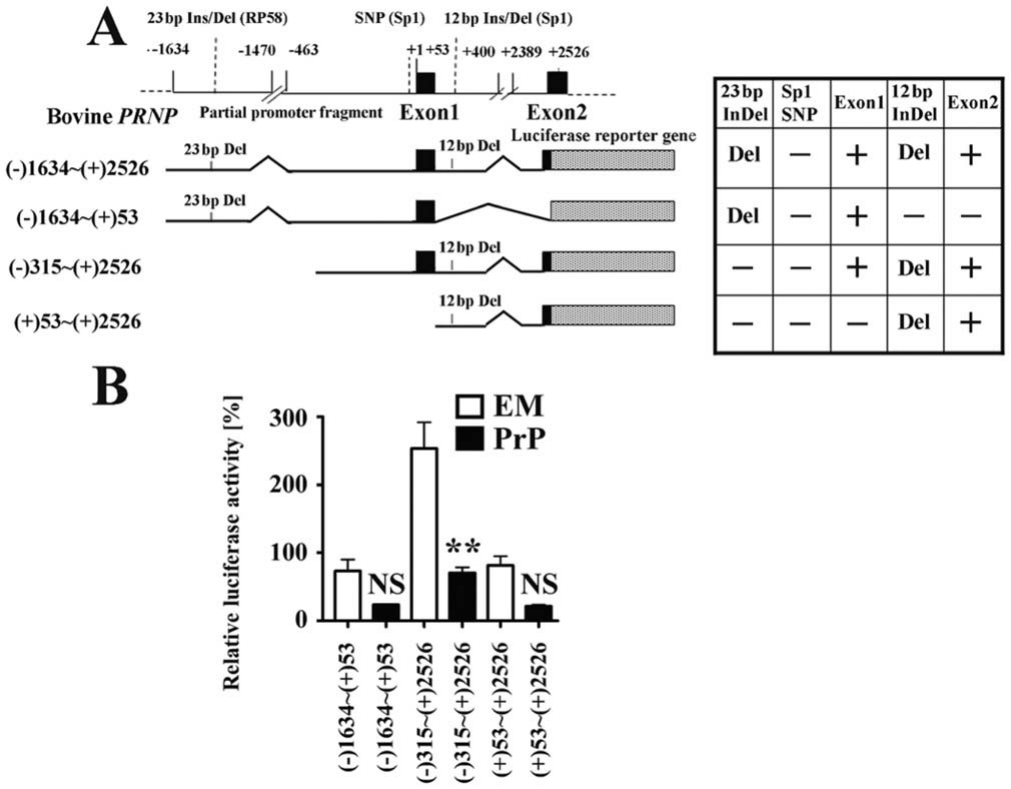
Deletion mutants show that the 23-bp deletion in the upstream region of *PRNP* and/or the 12-bp deletion in intron1, coupled with the absence of the Sp1 SNP and the presence of exon1, are required for the negative feedback response to PrP overexpression during regulation of prion protein expression. (A) Deletion mutants of the DelDel constructs as shown on the left were used. Dotted box = Luciferase gene; black boxes = exon 1 and exon 2, which include numbers denoting the position of the reported transcription start site (+1) of the *PRNP* promoter region. The 23-bp indel, 12-bp indel, and SNP regions are also indicated above the reporter gene constructs. The absence (−) and presence (+) of each region in the reporter gene constructs are shown in the Table on the right. (B) Graph representing relative luciferase activities obtained with the above reporter plasmids in the presence of either an empty vector or pEF-boPrP. Relative luciferase activities (Mean ± S.D.) for 3 replicate experiments were compared with the pGL3-control plasmid (1%). A significant difference of luciferase activity in pEF-boPrP-transfected cells as compared with corresponding empty vector-transfected cells is shown by two asterisks (**, <0.01). NS indicates no significant difference.

Next, to determine the minimal promoter region needed for response to PrP overexpression, we constructed several deletion constructs using the DelDel plasmid as a template (Fig. 3A). As shown in Fig. 3B, luciferase activity was significantly decreased by PrP overexpression in cells transfected with the plasmid incorporating nucleotides −315 to +2526, but not those incorporating nucleotides −1634 to +53 and +53 to 2526.

Taken together, these results suggest that the presence of a 23-bp deletion in the 5′ flanking region of *PRNP* and/or a 12-bp deletion in intron1, coupled with absence of the Sp1 SNP and the presence of exon1 of bovine *PRNP*, are responsible for negative feedback regulation of the PrP expression.

## Discussion

To investigate whether there is negative feedback regulation in *PRNP*, the effect of overexpression of bovine PrP on the bovine *PRNP* promoter region was analyzed. A luciferase-expressing vector (containing the *PRNP* promoter), bovine PrP expression vector pEF-boPrP, and a pRL-SV internal control plasmid were co-transfected into N2a cells. Promoter activity of *PRNP* was then analyzed.

In this study, the promoter activity of *PRNP* was inhibited by the overexpression of PrP, suggesting the presence of negative feedback regulation in *PRNP*. We used a previously constructed luciferase-expressing plasmid incorporating the DelDel allele, which contains a deletion of 23-bp in the upstream region of the *PRNP* promoter and a 12-bp deletion in intron 1 [9]. A 23-bp Ins/Del polymorphism containing the binding site for the transcription factor RP58 (Repressor protein with a predicted molecular mass of 58 kDa), and a 12-bp Ins/Del polymorphism containing the Sp1-binding site have been described in European Friesian cattle [10], whereas the polymorphisms −6→T, −47→A, −184A→G, −141T→C, −85T→G in the Sp1-binding site in the 5′-flanking region and +17→T and +43C→T in exon1 have been documented in Japanese black cattle [9],[11]. Here, the 23-bp-Ins and 12-bp-Ins allele showed lower expression levels of PrP as compared with the Del/Del allele. Our results are consistent with those described in previous reports [9], [10], [12]. In addition, nucleotides −88 to −30 within the 5′-flanking region and +123 to +891 in intron1 of the bovine PrP gene were found to be responsible for promoter activity.

A comparison of polymorphisms and their promoter activity showed that two Sp1-binding sites control Sp1 binding to the *PRNP* promoter and its activity, and the polymorphisms −6C→T, −47C→A, and −141T→C decreased PrP expression. The present study confirms the mechanism controlling basal expression of PrP described previously [9]. In addition, for the first time, we show that the 23-bp-Ins and 12-bp-Ins regions do not respond to negative feedback induced by PrP overexpression. Furthermore, in the presence of the Sp1 SNP, the DelIns allele is not downregulated by PrP overexpression. This suggests that the Sp1 SNP is able to function in collaboration with the above-described 23-bp and 12-bp Ins/Del polymorphisms in terms of negative feedback. Our deletion study supports this statement. In the absence of the Sp1 SNP and the presence of exon1, the presence of the Del in either the 23-bp Ins/Del or 12-bp Ins/Del region was required for the response to PrP overexpression. Nucleotides −315 to +2526, including the 5′-flanking region and exon1, are essential for the response. This sequence covers the region important for basic expression of PrP (−88 to −30 and +123 to +891).

This study also provides insight into the relationship between PrP expression and transcription factors. Analysis of polymorphisms within the bovine *PRNP* promoter supports the hypothesis that RP58 and Sp1 contribute to *PRNP* promoter activity [10]. In the present study, the 23-bp insertion and 12-bp insertion together did not show any response to PrP overexpression, whereas the wild-type Del/Del allele responded to PrP overexpression. This finding suggests that RP58 and Sp1 have a regulatory effect on PrP overexpression. The finding that intron 1 has its own promoter activity and contributes to the full activity of the gene sequence is in agreement with previous studies of both the murine and the bovine *PRNP* gene [13], [14]. In addition, intron 1 possibly plays a greater role in expression of the protein than that of just a regulator element. Intron 1 in isolation of the promoter has been shown to be sufficient to drive expression [15]. In the absence of intron 1, exon 1 inhibits promoter activity in most cell types studied [15], suggesting that *PRNP* has a unique regulatory structure in which sequences in intron 1 are the dominant elements in controlling expression.

These data are consistent with the findings that the promoter activity of the nucleotide regions −1634 to +53 and +53 to +2526 were decreased when compared with the region −315 to +2526, in which exon1 is combined with intron 1. The sequence in the region +65 to +155 contains binding sites for C/EBPα, NFY, and GATA-2, each of which has been shown to suppress promoter activity in other genes [16]–[18]. Therefore, such factors may also contribute to basic expression of PrP and negative feedback regulation by PrP overexpression. *PRNP* has a three-exon structure in all species except in humans and hamsters whose *PRNP* consists of two exons. The second exon in humans and hamsters may not be spliced into the final mRNA sequence. The third and final exon encodes the entire open reading frame (ORF) of the protein. There is also evidence for a splice variant in both cattle and mice that includes exons 2 and 3 but not exon 1. This indicates that exon 1 has another role. Candidate transcription activators that may bind to binding sites identified in exon 1 include Sp1, AP-2, and C/EBPα. The expression of many genes is regulated by elements within exon 1 [19], [20], one report shows that exon 1 may also be involved in *PRNP* regulation [15]. Proteins binding within the exon 1 region repress transcription of the *PRNP* promoter. Similar repression of promoter activity by transcription factors binding to sequences within exon 1 has been observed in other promoters [21]. Our data show that intron 1 and exon 1 are necessary for the response to PrP overexpression.

In conclusion, we show a negative feedback mechanism of PrP overexpression. Furthermore, a 23-bp deletion in the upstream region of *PRNP* and/or a 12-bp deletion in intron1, coupled with the absence of the Sp1 SNP and the presence of exon1, are required for the negative feedback response to PrP overexpression. This knowledge of the regulation of PrP expression may be useful in seeking an approach to reduce the risk of BSE.

## Materials and Methods

### Plasmids construction

The DelDel, DelIns, DelDel-Sp1, InsDel, InsIns, and InsIns-Sp1 promoter luciferase plasmids were described in our previous study [9]. The other three deletion mutants of promoter luciferase plasmid, covering nucleotides −1634 to +53, −315 to +2526, and +53 to +2526, were constructed by using plasmid DelDel as a template.

A mammalian expression plasmid for PrP was constructed by the following method. The ORF of bovine *PRNP* was amplified from Japanese black cattle genomic DNA from fat tissues by polymerase chain reaction (PCR) using the following primers: sense, 5′-AGctcgagATGGTGAAAAGCCACATAGGCAGT-3′; antisense, 5′-TCgcggccGCCTATCCTACTATGAGAAAAATGAG-3′. The lower-case letters indicate the restriction sequences of *Xho* I and *Not* I, respectively.

PCR products were cloned into a pT7BlueT-vector, sequenced using an ABI Prism 310 Genetic Analyzer (Applied Biosystems Inc., Foster City, CA, USA), and compared with the database sequences of bovine *PPNP* (Gene Bank: AJ298878). The plasmid containing the ORF of bovine *PRNP* was subsequently cloned into the vector pEF-BOS [22] to produce pEF-boPrP. The vector pEF-boPrP, which is a powerful mammalian expression vector that includes the human EF-α chromosomal gene, was used to overexpress PrP. The DNA sequences of the ORF of bovine *PRNP* in the pEF-BOS vectors were verified by sequencing using an ABI Prism 310 Genetic Analyzer.

### Cell culture and transfection

Neuroblastoma cells (N2a) [23], which was purchased from American Type Culture Collection (ATCC® Number: CCL-131™), were cultured in Eagle's minimum essential medium with nonessential amino acids and sodium pyruvate, and supplemented with 10% fetal calf serum at 37°C under 5% CO_2_ for the luciferase assay. For the promoter assays, N2a cells were seeded at 5×10^4^ cells/well in 24-well plates 48 h before transfection. Cells reaching 60–80% confluency were co-transfected with 540 ng of bovine *PRNP* promoter luciferase vector [9], 5 µg of bovine PrP expression vector (pEF-boPrP) or empty vector (pEF-BOS), and 60 ng of pRL-SV internal control plasmid. The transfections were carried out using Lipofectamine™ LTX and PLUS™ reagents according to the manufacturer's protocol (Invitrogen, Carlsbad, CA, USA).

### Recombinant bovine PrP and HRP labeling of mAb 1D12

Recombinant bovine PrP (25–241) (Alicon AG, Wagistrasse, Switzerland) was used. The monoclonal antibody (mAb) 1D12 [24] was labeled with horseradish peroxidase (HRP) using the Peroxidase-labeling Kit-SH (Dojindo, Kumamoto, Japan) according to the manufacturer's instructions.

### Enzyme-linked immunosorbent assay (ELISA)

Microtiter plates (Nunc-Immuno™ Modules; Nalge Nunc International, Rochester, NY, USA) were coated with 100 µl of anti-PrP mAb T2 [25] (1 µg/ml) in 0.1 M carbonate buffer (pH 9.5) overnight at 4°C and washed with 0.05% Tween20 in phosphate-buffered saline (PBS-T) three times. The coated plates were blocked 200 µl of Block Ace (diluted 1∶4 in PBS-T) for 1 h at room temperature and subsequently rinsed with PBS-T three times. 100 µl of samples diluted in phosphate-buffered saline (PBS) were added to the wells. The plates were incubated for 1 h at room temperature. The plates were washed with PBS-T five times before 100 µl of HRP-labeled anti-PrP mAb 1D12 (0.5 µg/ml) in PBS-T was added to the wells. After washing with PBS-T, 100 µl of o-phenylenediamine (Sigma-Aldrich Japan, Tokyo, Japan) solution was dispensed into each well. After incubating 30 min in the dark box, 20 µl of 6 N H_2_SO_4_ was added to the wells, and the absorbance was read at 490 nm on a Microplate reader (Bio-Rad, Hercules, CA, USA).

### Western blot assay

Cell pellets were suspended in radio-immunoprecipitation assay (RIPA) buffer composed of 10 mM Tris-HCl (pH 7.4) containing 1% sodium deoxycholate, 1% Nonidet P-40, 0.1% sodium dodecyl sulfate (SDS) and 0.15 M sodium chloride supplemented with 2 mM phenylmethylsulfonyl fluoride (PMSF), and then lysed in 2× SDS gel-loading buffer [90 mM Tris/HCl (pH 6.8), 10% mercaptoethanol, 2% SDS, 0.02% bromophenol blue, 20% glycerol]. The samples were boiled for 5 min before an equal quantity of protein (20 µg) was subjected to electrophoresis on SDS/12% polyacrylamide gels. Proteins electrically transferred onto polyvinylidene difluoride (PVDF) membrane (Hybond-P; Amersham-Pharmacia Biotech, Piscataway, NJ, USA) were treated by BLOCK-ACE (Dainippon pharmaceutical, Osaka, Japan) at 4°C overnight. Membranes were then incubated in PBS containing 0.1% Tween-20 (PBS-TW) and 10% BLOCK-ACE for 1 h at room temperature with one of the following anti-PrP antibodies: SAF32 or 6H4 (Prionics, Zürich, Switzerland) [26]. As a loading control, antibody α-tubulin B-5-1-2 (Sigma-Aldrich Japan, Tokyo, Japan) was used. After washing with PBS-TW, the membrane was incubated in secondary antibody, HRP-conjugated anti-mouse IgG (Jackson ImmunoResearch Laboratories, Inc., West Grove, PA, USA), for 1 h at room temperature. After three washes in PBS-TW, the probed proteins were detected using an enhanced chemiluminescence detection kit (Amersham-Pharmacia Biotech).

### Luciferase assay

The luciferase activity of cell lysates prepared at 48 h after transfection was measured as relative light units with the TriStar LB 941 Multimode Reader (Berthold Technologies, Bioanalytic, Bad Wildbad, Germany) using the Dual-Luciferase Assay System (Promega, Madison, WI, USA). Relative luciferase activities were defined as the ratio of the firefly luciferase activity to mean total protein value of each construct related to the pGL3-control Vector, which contains the SV40 promoter.

### Statistical analysis

Before applying statistical analysis for comparison of groups, we analyzed the data using the D'Agostino-Pearson test. This is because it is essential to determine how far from Gaussian distribution the data is in terms of asymmetry and shape in order to choose the most suitable method of statistical analysis. Specifically, the D'Agostino-Pearson test was performed to check whether groups showed parametric data, which means Gaussian distribution. Statistical analysis of two groups was performed using the unpaired *t* test for parametric data and the Mann-Whitney test for nonparametric data [27]. Statistical analysis of more than three groups should be performed using one-way analysis of variance followed by the Bonferroni test for parametric data, and the Kruskal–Wallis test followed by Dunnett's multiple comparison test for nonparametric data. In the analyses, two-tailed asymptotic significance levels were considered. P values less than 0.05 were considered significant. Calculations were performed using GraphPad Prism 4 (GraphPad software, Inc., San Diego, CA, USA).

## Supporting Information

Figure S1 **Investigation of absolute transfection efficiency by green fluorescent protein (GFP).** (A, B) To investigate absolute transfection efficiency, a green fluorescent protein (GFP) expression vector was transfected into Neuro-2a (N2a) cells. Forty-eight hours after transfection, the N2a cells were analyzed by fluorescence microscopy. A high transfection efficiency of GFP was observed. (C, D) Negative control. Scale bar is 50 µm.
(TIFF)

Figure S2 **Detection of bovine PrP in N2a cells by sandwich ELISA.** Expression of bovine PrP in N2a cells after transfection of pEF-boPrP (0–10 µg) was detected by sandwich ELISA with T2 as a capture mAb and 1D12-HRP as a detection mAb. Each data point is the mean of three determinations.
(TIFF)

## References

[1] Sakudo A, Ikuta K (2009). Prion protein functions and dysfunction in prion diseases.. Curr Med Chem.

[2] Prusiner SB (1998). Prions.. Proc Natl Acad Sci U S A.

[3] Bueler H, Aguzzi A, Sailer A, Greiner RA, Autenried P (1993). Mice devoid of PrP are resistant to scrapie.. Cell.

[4] Mallucci G, Dickinson A, Linehan J, Klohn PC, Brandner S (2003). Depleting neuronal PrP in prion infection prevents disease and reverses spongiosis.. Science.

[5] Fischer M, Rulicke T, Raeber A, Sailer A, Moser M (1996). Prion protein (PrP) with amino-proximal deletions restoring susceptibility of PrP knockout mice to scrapie.. EMBO J.

[6] Sakudo A, Onodera T, Suganuma Y, Kobayashi T, Saeki K (2006). Recent advances in clarifying prion protein functions using knockout mice and derived cell lines.. Mini Rev Med Chem.

[7] Sakudo A, Ikuta K (2009). Fundamentals of prion diseases and their involvement in the loss of function of cellular prion protein.. Protein Pept Lett.

[8] Ishiura M, Kutsuna K, Aoki S, Iwasaki H, Andersson CR (1998). Expression of a gene cluster kaiABC as a circadian feedback process in cyanobacteria.. Science.

[9] Xue G, Sakudo A, Kim CK, Onodera T (2008). Coordinate regulation of bovine prion protein gene promoter activity by two Sp1 binding site polymorphisms.. Biochem Biophys Res Commun.

[10] Sander P, Hamann H, Drogemuller C, Kashkevich K, Schiebel K (2005). Bovine prion protein gene (*PRNP*) promoter polymorphisms modulate *PRNP* expression and may be responsible for differences in bovine spongiform encephalopathy susceptibility.. J Biol Chem.

[11] Nakamura I, Xue G, Sakudo A, Saeki K, Matsumoto Y (2007). Novel single nucleotide polymorphisms in the specific protein 1 binding site of the bovine *PRNP* promoter in Japanese Black cattle: impairment of its promoter activity.. Intervirology.

[12] Msalya G, Shimogiri T, Ohno S, Okamoto S, Kawabe K (2011). Evaluation of PRNP expression based on genotypes and alleles of two indel loci in the medulla oblongata of Japanese Black and Japanese Brown cattle.. PLoS One.

[13] Inoue S, Tanaka M, Horiuchi M, Ishiguro N, Shinagawa M (1997). Characterization of the bovine prion protein gene: the expression requires interaction between the promoter and intron.. J Vet Med Sci.

[14] Premzl M, Delbridge M, Gready JE, Wilson P, Johnson M (2005). The prion protein gene: identifying regulatory signals using marsupial sequence.. Gene.

[15] Haigh CL, Wright JA, Brown DR (2007). Regulation of prion protein expression by noncoding regions of the *Prnp* gene.. J Mol Biol.

[16] Timchenko NA, Wilde M, Nakanishi M, Smith JR, Darlington GJ (1996). CCAAT/enhancer-binding protein alpha (C/EBP alpha) inhibits cell proliferation through the p21 (WAF-1/CIP-1/SDI-1) protein.. Genes Dev.

[17] Desaint S, Hansmannel F, Clemencet MC, Le Jossic-Corcos C, Nicolas-Frances V (2004). NFY interacts with the promoter region of two genes involved in the rat peroxisomal fatty acid beta-oxidation: the multifunctional protein type 1 and the 3-ketoacyl-CoA B thiolase.. Lipids Health Dis.

[18] El Wakil A, Francius C, Wolff A, Pleau-Varet J, Nardelli J (2006). The GATA2 transcription factor negatively regulates the proliferation of neuronal progenitors.. Development.

[19] Kim Y, Lee J, Lee C (2008). In silico comparative analysis of DNA and amino acid sequences for prion protein gene.. Transbound Emerg Dis.

[20] Sakudo A, Xue G, Kawashita N, Ano Y, Takagi T (2010). Structure of the prion protein and its gene: an analysis using bioinformatics and computer simulation.. Curr Protein Pept Sci.

[21] Aoki K, Meng G, Suzuki K, Takashi T, Kameoka Y (1998). RP58 associates with condensed chromatin and mediates a sequence-specific transcriptional repression.. J Biol Chem.

[22] Mizushima S, Nagata S (1990). pEF-BOS, a powerful mammalian expression vector.. Nucleic Acids Res.

[23] Windl O, Lorenz H, Behrens C, Römer A, Kretzschmar HA (1999). Construction and characterization of murine neuroblastoma cell clones allowing inducible and high expression of the prion protein.. J Gen Virol.

[24] Hosokawa T, Tsuchiya K, Sato I, Takeyama N, Ueda S (2008). A monoclonal antibody (1D12) defines novel distribution patterns of prion protein (PrP) as granules in nucleus.. Biochem Biophys Res Commun.

[25] Shimizu Y, Kaku-Ushiki Y, Iwamaru Y, Muramoto T, Kitamoto T (2010). A novel anti-prion protein monoclonal antibody and its single-chain fragment variable derivative with ability to inhibit abnormal prion protein accumulation in cultured cells.. Microbiol Immunol.

[26] Korth C, Stierli B, Streit P, Moser M, Schaller O (1997). Prion (PrPSc)-specific epitope defined by a monoclonal antibody.. Nature.

[27] Miller JN, Miller JC (2010). Statistics and chemometrics for analytical chemistry.

